# Forehead against Resistance (FAR): Preliminary Findings from A Clinical Alternative to Shaker's Type of Exercise

**DOI:** 10.1155/2019/9387578

**Published:** 2019-03-24

**Authors:** Radish Kumar Balasubramaniam, Rahul Krishnamurthy, Athira Rajan, Suprasanna K

**Affiliations:** ^1^Department of Audiology and Speech-Language Pathology, Kasturba Medical College, Mangalore, India; ^2^Department of Radiodiagnosis, Kasturba Medical College, Mangalore, India; ^3^Manipal Academy of Higher Education, Manipal, Karnataka, India

## Abstract

**Background:**

Reduced UES opening is a well-known risk factor for dysphagia. The Shaker exercise and the CTAR are the widely used intervention strategies to bring about effective UES opening. But there are well-known difficulties with the clinical use of these two exercise regimes. The present study proposes a clinical alternative to Shaker's exercise and CTAR called the forehead against resistance (FAR) and its variants without altering the central principles of these two regimes. The aim of the present study was to investigate the efficacy of FAR and its variants in bringing about UES opening.

**Method:**

The study used a comparative cross-sectional study design, with the nonrandomized convenient sampling that included 27 healthy adults. MBS was carried out in the anterior-posterior and lateral views, while the participants performed FAR and its variants. The UES diameter was measured in the baseline and with the subjects performing FAR maneuver and its variants.

**Results:**

The result revealed that the participants had greater UES opening on FAR and its variant than the baseline swallow. Also, mean values of UES opening were greater for FAR with chin tuck when compared to FAR alone, although there was no significant main effect with exercise.

**Conclusion:**

FAR and its variant could be one of the options for increasing UES opening in individuals with dysphagia.

## 1. Introduction

The integrity of the pharyngeal phase of the swallow is determined by an adequate opening of the upper esophageal sphincter (UES) that conduits smooth transit of bolus. Reduced UES opening is a known risk factor for dysphagia, although the actual prevalence of dysphagia secondary to reduced UES opening is not well established [1]. The physiological act of opening of the UES is determined by four primary factors (for an overview, see [2]); out of these, only the suprahyoid contraction is accessible to direct treatment. Among the existing dysphagia intervention strategies, the Shaker exercise (see [3]) and chin tuck against resistance (CTAR) exercises (see [4]) have been designed explicitly to target the UES opening by strengthening the suprahyoid muscles.

Efficacy of Shaker's exercise has been well tested and has proven to be an effective regime in treating dysphagia secondary to reduced UES opening [5–7]. However, studies have also reported adverse effects, such as muscle discomfort and time consumption [8] and also being physically demanding for the elderly [9]. To overcome this, Yoon et al. [4] proposed a modified variant of Shaker's exercise called the chin tuck against resistance (CTAR). It is aimed at effectively activating the suprahyoid muscles while not being strenuous as that of Shaker's regime. A recent study by Sze et al. [1] has reported CTAR to be an effective regime in achieving UES opening.

We have routinely used both the above-discussed regimes for the treatment of dysphagia secondary to reduced UES opening, and our anecdotal evidence suggests that both these regimes are useful on a clinical basis. However, our clinical use of these regimes on a regular basis was faced with several challenges. Foremost, Shaker's regime was perceived by the patients to be physically demanding, time-consuming, and difficult to comprehend which are in agreement with existing literature [8, 9]. Secondly, as reported by Sze et al. [1], factors such as diameter and hardness properties of the ball used, suitability of the ball based on the neck anthropometry of each individual, remain to pose a clinical challenge.

These inherent difficulties necessitate designing of specific therapeutic strategies that could achieve the opening of UES while overcoming the drawbacks of existing ones. One such possibility is modifying the procedures of Shaker's regime and CTAR while respecting their central principles. Considering this, we propose a modified variant to Shaker's type of exercise called the forehead against resistance (FAR) maneuver. The resistance offered during Shaker's exercise is due to the head lifts against gravity. For the FAR exercise, the resistance is offered by the clinician pressing his palm against the patient's forehead while being seated. Simultaneously, the patient is asked to swallow while applying a forward push to the clinician's palm. We hypothesize that the head and neck trajectory achieved by FAR will mirror that in Shaker's exercise even though the FAR maneuver is performed being seated rather than lying down, thus providing a possible clinical alternative to the traditional form.

Chin tuck is another widely used strategy for activating suprahyoid and infrahyoid muscles [10, 11]. Considering this, we proposed the second variant called the forehead against resistance with chin tuck. We hypothesize that adding a component of chin tuck to FAR ensures that there is an evident involvement of suprahyoid and infrahyoid muscles during the maneuver. This is done with the intention to overcome the shortcomings of CTAR [4] while respecting its founding principles.

However, the efficacy of these FAR maneuvers requires to be tested on healthy individuals before it can be used on the clinical population. Thus, the present study was taken up to investigate questions like the following. To what extent do these two exercise regimes facilitate UES opening? Can the relative changes in UES opening be quantified by a gold standard such as Modified Barium Swallow (MBS)? Therefore, the aim of the study was to investigate the immediate effects of FAR and FAR with chin tuck on UES opening as measured on MBS among typical adult individuals. We also hypothesize that there would be no difference in UES opening between FAR and FAR with chin tuck.

## 2. Method

### 2.1. Study Design

The study used a comparative cross-sectional design with nonrandomized convenient sampling.

We further adopted a repeated measure strategy with the order of two exercises counterbalanced across two variants and participants. The order effect of these exercises was minimized by using the Latin square method.

### 2.2. Participants

Prior to the recruitment of participants, the study was approved by the local Institution Review Board (IEC KMC MLR 10-16/268). A total of 27 normal adult participants in the age range of 18 to 30 were considered for the study based on the sample size formula 2(*Z*_*α*_ + *Z*_*β*_)^2^*σ*^2^/*d*^2^, *Z*_*α*_ = 1.96, at 95% confidence intervals. Prior to the MBS procedure, all the participants were screened using the Manipal Manual of Swallowing Assessment (MMSA) (Balasubramaniam and Bhat, 2012). Only those individuals with a score of “0” on MMSA (0 indicates normal swallowing functions) were considered for the study. Further, these individuals were ruled out for speech, language, and swallowing disorders; neurological, metabolic, and systemic disorders (hypertension and diabetes mellitus); and use of artificial dentures. None of the individuals had undergone any surgery to the oropharyngeal apparatus in the past.

### 2.3. Procedure

**Figure 1:**
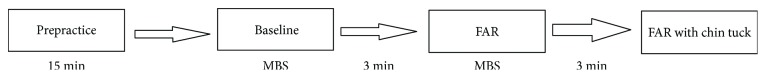
The series of steps involved in the recording procedure.

The entire procedure was carried out in the following steps and has been depicted in Figure 1.

#### 2.3.1. Prepractice Sessions

The present study required normal individuals to be familiarized and trained for each variant of the exercise regimes. All the participants were given a practice session of 15 minutes, where they were familiarized with both the exercises. Steps involved in each of the exercise regimes were explained and demonstrated by the first author. Following this, all the participants practiced each of the exercises under the supervision of the first author for a span of 15 minutes. Once the participants were familiarized and trained for both the regimes, they received a break time of 15 minutes before the baseline MBS recordings were made.

#### 2.3.2. Baseline Recording

All the participants ingested the given 10 ml of liquid thin barium (E-Z-HD Barium sulfate powder for suspension and water/50–50; approximately 14 cP) delivered through a measuring cup. They were instructed to hold the bolus in their mouth and swallow upon receiving the verbal cue “swallow.” All the participants swallowed the entire bolus in a single attempt. The baseline MBS recording was obtained at 90 keV, using a 9-inch image intensifier mode and appropriate collimation so that an image was obtained of the posterior mouth, pharynx, and pharyngoesophageal region. The images were obtained in anterior-posterior and lateral views with 30 frames/60 fields/s when the participants were standing on the platform attached to the fluoroscopic table. The volume and consistency of barium (10 ml liquid thin) were kept constant across further steps.

#### 2.3.3. Forehead against Resistance

**Figure 2:**
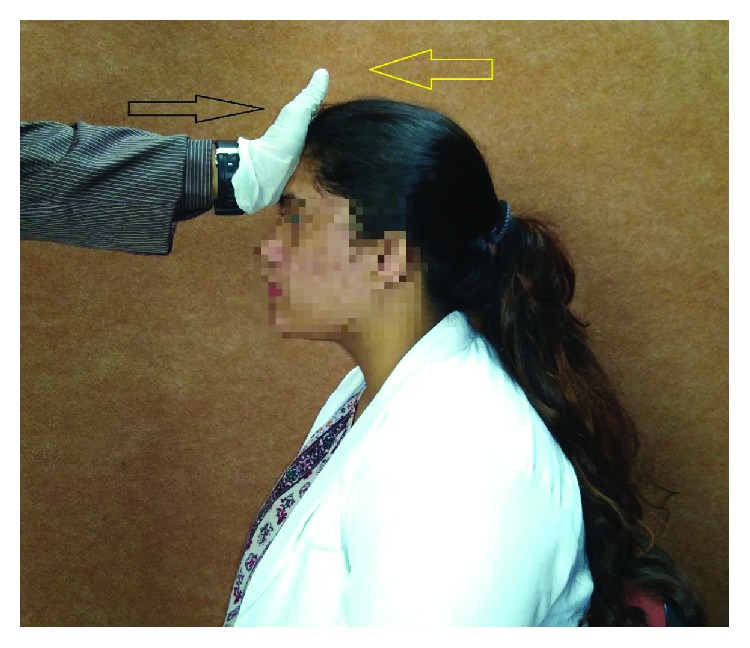
Steps involved in forehead against resistance (FAR).

The participants were instructed to swallow while performing FAR maneuver (as depicted in Figure 2), and during this, a second MBS recording was obtained simultaneously in anterior-posterior and lateral views with the participant performing the maneuver. Following this, the diameter of UES opening was measured, and the participants were given one minute of rest before the commencement of the next exercise regime.

#### 2.3.4. Forehead against Resistance with Chin Tuck

**Figure 3:**
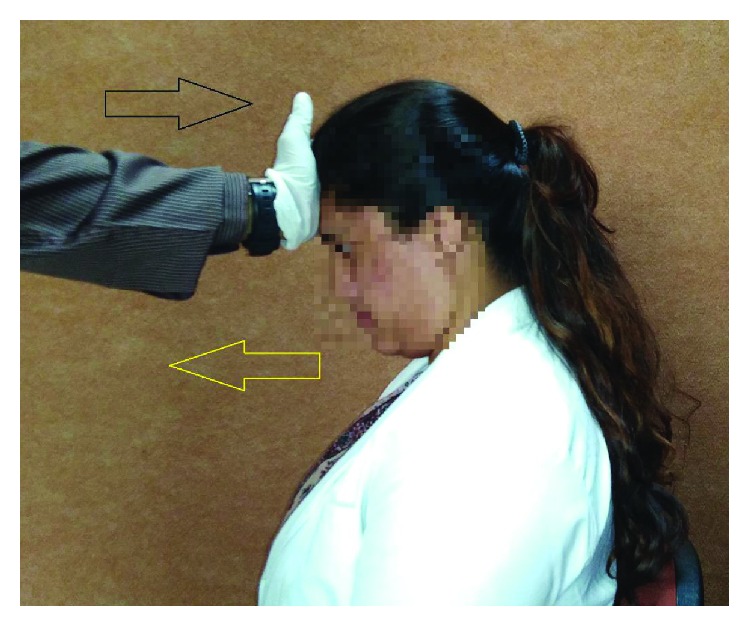
Steps involved in forehead against resistance (FAR) with chin tuck.

Here, the participants were instructed to ingest the liquid barium by keeping chin down towards manubrium sterni and swallow while performing the FAR maneuver (as depicted in Figure 3). During this, a third MBS recording was carried out, and the opening of the upper esophageal sphincter was measured in both anterior-posterior and lateral views.

### 2.4. Data Analysis

The upper esophageal sphincter diameter was measured using the X-ray image when the liquid barium passes between the cervical 5 (C5) and cervical 6 (C6) vertebrae. Each X-ray image was submitted to the ImageJ 1.32j program and was rotated to a true 90 degrees to make calculations. The distance between the anterior corners (superior and inferior) of C3 was used as the known length (15 mm) to mark points to actual size. The diameter between the C5 and C6 was marked in the anterior-posterior swallow position as well as in the lateral position within the ImageJ 1.32j program. The measurement procedure followed the same lines for both the anterior-posterior view and the lateral view. The values of all the measures were tabulated in the Microsoft Excel sheet. Statistical analysis was done using the SPSS, version 17.0. One-way mixed ANOVA was performed with the variant of exercise as the within-group variable and gender as the group variables.

## 3. Results

From Table 1, it can be observed that there were no clinical differences on UES opening across the two exercise regimes. Gender differences were also not clinically significant. One-way mixed ANOVA was performed with exercise as the within-group variable and gender as the between-group variable. The result revealed no significant main effect for exercise (*F*_(2, 23)_ = 1.591, *p* = 0.225) and gender (*F*_(2, 23)_ = 0.596, *p* = 0.596). There was no interaction effect between variants of exercise and gender (*F*_(2, 48)_ = 0.699, *p* = 0.502).

Since the significant main effect of exercise was not observed, qualitative analysis of the raw data was performed to see the individual variations. While comparing the UES diameter, in the A-P view, between the baseline and FAR, 6 subjects showed an increment in the diameter, 5 subjects showed a decrement in diameter, and 15 subjects showed apparently no change. On inspection of the lateral view, 7 subjects showed increased UES opening, 3 subjects showed decreased UES opening, and 16 subjects showed relatively no change.

On comparing the UES diameter, between the baseline and FAR with chin tuck, in the A-P view, 5 subjects showed an increment in the diameter, 6 subjects showed a decrement in diameter, and 15 subjects showed apparently no change. For the lateral view, 4 subjects showed increased UES opening, 1 subject showed decreased UES opening, and 21 subjects showed relatively no change. It was also interesting to note that participants who showed improved diameter with both FAR and FAR with chin tuck in the lateral view did not obtain similar findings in the anterior-posterior view except for one participant who showed improved UES diameters in both the views.

### 3.1. Feedback from Participants

From the subjective feedback obtained from all the participants on ease of use and physical demand, 25 out of 27 participants reported that both the variants were not strenuous.

## 4. Discussion

The descriptive data from the present study reveal that FAR and its variant resulted in slightly greater UES opening than that of baseline measure. Although no statistical significance was revealed, our observations revealed that the mean values of UES diameter were slightly higher when FAR with chin tuck was performed rather than FAR alone. This indicates the fact that, even though statistically significant results could not be obtained, FAR with chin tuck could be one of the options for increasing UES opening in individuals with dysphagia. This finding can be attributed to the combined effect of cricopharyngeal muscle's relaxation and distensibility and also to retraction of the cricoid cartilage which is caused by contraction of the group of the suprahyoid muscles in UES opening [3].

A probable reason for no significant difference in the UES opening across the variants could be due to the lack of repeated trials with the exercises. In the present study, both the variants of FAR were introduced as a maneuver for immediate use rather than an exercise regime. We contemplate that if the proposed variants are used in the form of an exercise regime, there could be a more significant impact on the overall physiology of upper esophageal sphincter opening which could be statistically measured. The failure of FAR and FAR with chin tuck to demonstrate a statistically significant increment among healthy adult individuals may probably be due to intact central control of swallowing and intact suprahyoid muscles and normal UES opening. This lack of increment in healthy adult individuals does not preclude a potential benefit in patients with neurogenic dysphagia who have demonstrable reduction in UES opening.

An interesting finding from the present study is that all the participants subjectively report both the variants to be not strenuous. Even though objective measures of effort were not implemented in the preliminary study, the subjective findings reinforce the clinical feasibility of the FAR and its variant.

## 5. Limitations and Future Directions

The preliminary findings from the present study are encouraging, but few known limitations of the study need to be acknowledged. First, both the variants were used as a maneuver to investigate their immediate effects on UES opening. Future studies should consider the use of the proposed variants in the form of exercise regimes and investigate its effects on UES opening. Secondly, the participants considered in the present study were healthy adults; the feasibility of the proposed maneuver among geriatrics and disordered population requires further investigation. Appropriate care should be taken to assess the safety of these maneuvers among these populations.

## 6. Conclusion

The present study was a preliminary attempt towards designing a clinical alternative to Shaker's exercise and CTAR. It is aimed at investigating the efficacy of the proposed maneuver on the actual opening of UES in healthy subjects. The result revealed that the participants had greater UES opening on FAR and its variant compared to the baseline swallow. FAR and its variant could be one of the options for increasing UES opening in individuals with dysphagia.

## Figures and Tables

**Table 1:** Mean and SD of upper esophageal opening diameter using modified Shaker's and CTAR in lateral and anterior-posterior views.

View	Baseline mean (SD)	FAR mean (SD)	FAR with chin tuck mean (SD)
Lateral view			
Male	7.65 (2.28)	8.72 (2.07)	9.01 (1.75)
Female	8.91 (2.44)	9.03 (3.46)	9.34 (2.94)
A-P view			
Male	18.3 (3.25)	17.13 (3.54)	17.46 (4)
Female	16.81 (2.63)	17.65 (2.88)	16.88 (2.92)

## Data Availability

The videofluoroscopic swallow study data used to support the findings of this study are restricted by the institutional ethical committee of the Kasturba Medical College Hospital, Mangalore, in order to protect participant privacy. Data are available from Dr. Radish Kumar who can be mailed at radheesh.slp@manipal.edu for researchers who meet the criteria for access to confidential data.
